# Safety of Live Immunization in DiGeorge Syndrome: A Retrospective Single-Center Study in Korea, 2005–2021

**DOI:** 10.3390/vaccines10122165

**Published:** 2022-12-16

**Authors:** Sung Min Lim, Je Hee Shin, Jee Yeon Baek, Ji Young Lee, Ji-Man Kang, Jong Gyun Ahn

**Affiliations:** 1 Department of Pediatrics, Severance Children’s Hospital, Yonsei University College of Medicine, Seoul 03722, Republic of Korea; 2 Institute for Immunology and Immunological Disease, Yonsei University College of Medicine, Seoul 03722, Republic of Korea

**Keywords:** live immunization, DiGeorge syndrome, adverse events, vaccination, immunodeficiency

## Abstract

Live immunization is contraindicated in patients with DiGeorge syndrome (DGS). We retrospectively investigated the occurrence of adverse events after live immunization in patients with DGS in Korea. The data of patients matching the International Classification of Disease-10 code of DGS (D82.1) at Severance Hospital Seoul, Korea, were extracted; patients without genetically diagnosed DGS were excluded. Based on T cell immunity status, the included patients were categorized into group A (CD3 < 500 or CD8 < 200 cells/mm^3^); group B (CD3 ≥ 500 and CD8 ≥ 200 cells/mm^3^); or group C (unknown). Among 94 patients, 38 (~40%, group A: 8 [21%]; group B: 30 [79%]) underwent immunological testing and 73 (~80%) received at least one live immunization (measles–mumps–rubella vaccination was most common [66/94, ~70%]). Fifty adverse events (fever [*n* = 29], upper respiratory infection [*n* = 9], diarrhea [*n* = 4], rash [*n* = 3], thrombocytopenia [*n* = 3], injection site pus [*n* = 1], and febrile convulsion [*n* = 1]) were observed; 13 (26%) occurred in group A, with no significant difference in incidence between groups A and B. Serious adverse events, including intensive care unit hospitalization or death, or diseases due to vaccine strains were not observed. In this study, live immunization was well tolerated by patients with partial DGS.

## 1. Introduction

DiGeorge syndrome (DGS), also known as 22q11.2 deletion syndrome, is the most common microdeletion syndrome, with a prevalence of 1 in 4000 live births [1]. It is characterized by a wide range of clinical features, including congenital heart disease, hypoparathyroidism, facial dysmorphism, developmental delay, and immunodeficiency [1,2,3]. Patients with DGS show varying levels of T cell deficiencies (ranging from an absence of T cells to normal counts) and percentages due to thymic hypoplasia [4]. A very low T cell count (CD3 < 50 cells/mm^3^ or < 1–2%) is often regarded as complete DGS, whereas patients with partial DGS (pDGS) contain relatively higher T cell counts with a wide range of lymphoproliferative functions [5,6].

Adverse events (AEs) following live immunization (AEFLI) in patients with primary immunodeficiency diseases, such as vaccine-acquired measles, Bacillus Calmette–Guérin (BCG), and rotavirus infection, have been extensively studied [7,8,9,10]. Further, several studies have reported severe AEFLI, such as septicemia and pneumonia, following live attenuated varicella (VAR) vaccination [11,12], and serious BCGitis after BCG vaccination in patients with DGS [9]. Hence, in previous guidelines, live immunization was contraindicated for patients with T cell deficiencies, including DGS [13]. However, studies have indicated that live vaccinations, such as measles–mumps–rubella (MMR) and VAR vaccines, are relatively safe and well-tolerated in patients with pDGS [14,15,16].

The recent Infectious Disease Society of America (IDSA) and American Academy of Pediatrics (AAP) recommendations indicate that MMR and VAR vaccines should be considered if a patient with DGS has adequate T cell counts (≥ 500 and ≥ 200 CD3 and CD8 T cells/mm^3^, respectively) and exhibits normal mitogen response. However, patients with DGS with CD3 T cell counts < 500 cells/mm^3^ should not be administered any live vaccine [17,18].

Studies on the identification of AEs after BCG, rotavirus, and live-attenuated Japanese encephalitis virus (LJEV) vaccinations in patients with pDGS are limited, probably because BCG and LJEV are rarely included in the National Immunization Program (NIP) worldwide. In Korea, however, BCG and LJEV vaccines are included in the NIP, along with MMR and VAR vaccines. According to the 2018 Korea Disease Control and Prevention Agency report, the vaccination completion rate for that year was >95% for BCG, MMR, and VAR, and >90% for LJEV [19]. Although the rotavirus vaccine is not included in the NIP, a complete immunization rate of > 85% has been reported [20]. Such high vaccination coverage rates allow AE evaluation following various types of live immunizations in patients with DGS in Korea. Furthermore, the Korean Pediatric Society recommends consideration of MMR and VAR vaccines if a patient with DGS has adequate T cell counts (≥ 500 and ≥ 200 CD3 and CD8 T cells/mm^3^, respectively) and exhibits a normal mitogen response [21], similar to global guidelines. However, investigation of AEFLI in patients with DGS in Korea is limited. Hence, this study was conducted to examine AEFLI in patients with DGS in Korea.

## 2. Materials and Methods

### 2.1. Patient Characteristics

This retrospective study included patients diagnosed with DGS at Severance Hospital, Seoul, Korea, between 1 November 2005 and 31 July 2021. The data of patients matching the International Classification of Disease-tenth revision (ICD-10) code of DGS (D82.1) were extracted from the Severance Clinical Research Analysis Portal. Patients lacking genetic confirmation of 22q11.2 deletions were excluded. The patients' clinical information was collected by reviewing medical charts, while the immunization records were obtained from the NIP electronic record. In this study, data on five vaccines—namely BCG, rotavirus, MMR, VAR, and LJEV—were included. Demographic characteristics (age and sex); medical history (genetic testing and cardiac history); laboratory findings (all available blood test results, including lymphocyte subsets, complete blood counts, hormones, and electrolytes); and imaging findings (echocardiogram and cardiac computed tomography [CT]) were collected.

The patients were categorized into the following groups based on the current vaccination guidelines of the IDSA, AAP, and Korean Pediatric Society [17,18,21]: (1) group A, (CD3 < 500 or CD8 < 200 T cells/mm^3^) contraindicated for live vaccination; (2) group B, (CD3 ≥ 500 and CD8 ≥ 200 T cells/mm^3^) recommended for MMR and VAR vaccinations; and (3) group C, unknown immunity status. This study was approved by the Institutional Review Board of Severance Hospital (IRB number: 2021-2423-003), and the requirement for informed consent was waived due to the retrospective nature of the study.

### 2.2. Definition of AEFLI

An adverse event following immunization is defined by the World Health Organization (WHO) as “any untoward medical occurrence which follows immunization, and which does not necessarily have a causal relationship with the usage of the vaccine.” Moreover, serious AEs following immunization include any event resulting in death; life-threatening condition; persistent or significant disability/incapacity; requirement for intervention to prevent permanent impairment or damage; requirement for in-patient hospitalization; prolongation of existing hospitalization; or induction of a congenital anomaly/birth defect [22]. In this study, we adapted the above-mentioned definition of AEFLI. To investigate AEFLI, we reviewed the outpatient department and emergency room visits, hospitalizations, and medical records within 60 days (12 months for BCG vaccination) from the date of live vaccination. We defined serious AEFLI according to the WHO definition [22], excluding the requirement for in-patient hospitalization or prolongation of existing hospitalization, as the study was retrospective in nature and hospitalized patients were already selected as study participants.

The collected AEFLI data were analyzed according to the WHO assessment models. The 1999 WHO classification for adverse reactions includes six categories: very likely, probable, possible, unlikely, unrelated, and unclassifiable adverse reactions [23]. Additionally, the 2019 WHO causality classification includes the following: (A) consistent causal association to immunization, (B) indeterminate, (C) inconsistent with causal association to immunization, and (D) unclassifiable through an algorithm [24].

### 2.3. Statistical Analysis

Fisher’s exact test was used to identify any associations between immune status and AEFLI, and a *p* value of < 0.05 was considered statistically significant. All data analyses were performed using SPSS software for Windows, version 25.0 (SPSS Inc., Chicago, IL, USA).

## 3. Results

### 3.1. Study Population

**Figure 1:**
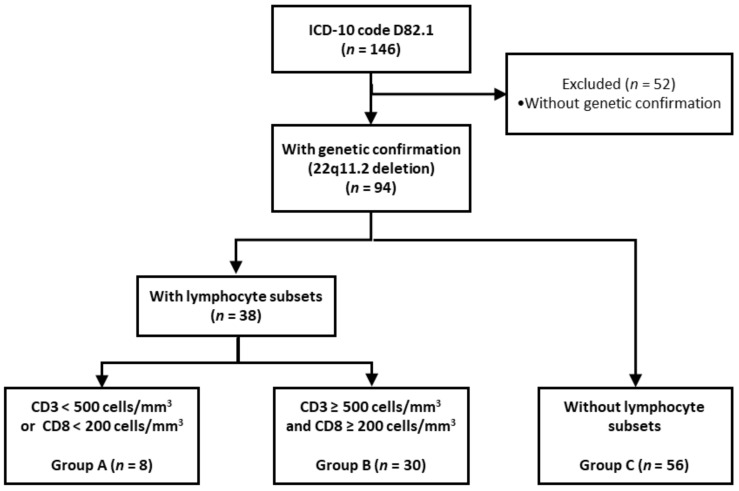
Flow chart of the study. Abbreviations: ICD-10, International Classification of Disease-10.

During the study period, a total of 146 patients with the ICD-10 code D82.1 were identified. Of these, 94 patients with confirmed 22q11.2 deletion were selected as study participants (Figure 1). Table 1 shows the baseline characteristics of the participants. Fifty-one patients (54%) were male, and the genetic analysis was mostly (57%) performed using fluorescence in situ hybridization, with a median age of 7.9 months at genetic confirmation (interquartile range [IQR], 0.7–104.3). Moreover, 30 patients (32%) were treated for hypocalcemia; 69 (73%) presented with congenital heart diseases, including ventricular septal defect (*n* = 25), tetralogy of Fallot (*n* = 22), and interrupted aortic arch (*n* = 9); and 61 underwent surgery. Other common clinical features included facial dysmorphism (*n* = 50 [53%]), developmental delay (*n* = 38 [40%]), and epilepsy (*n* = 6 [6%]).

CD3 and CD8 T cell counts were available for approximately 40% (38/94) of patients; these patients had a median age of 2.8 months (IQR 1.0–75.5) when tested, and none of them had complete DGS (8 [21%] and 30 [79%] patients belonged to group A and B, respectively; Figure 1). Lymphocyte proliferation testing data were available for two patients, of which only one showed normal values. Furthermore, thymus imaging data based on cardiac CT or thoracic sonography were available for 28 patients (30%), of which 11 (27%) appeared to have a relatively small-sized thymus, while the remaining patients had a normal-sized thymus (Table 1).

### 3.2. Immunization Coverage

Table 2 shows immunization coverage among the study participants. Of the 94 participants, approximately 80% (73/94) received at least one live vaccination. Further, MMR vaccination accounted for the highest number of vaccinations (66/94 [70%]), followed by VAR (47/94 [50%]), BCG (45/94 [48%]), rotavirus (23/94 [24%]), and LJEV (18/94 [19%]) vaccinations. Approximately half of the vaccinated patients (36/73 [49%]) received a live vaccine before confirmation of 22q11.2 deletion.

In group A, 6 of the 8 (75%) patients received at least one live vaccination, with a total of 29 doses; half of these were vaccinated before diagnosis or lymphocyte subset analysis. MMR and VAR vaccination rates were the highest (63%, 5/8), followed by BCG (50%, 4/8), LJEV (38%, 3/8), and rotavirus (13%, 1/8) vaccination. In group B, 24 of the 30 (80%) patients had at least one live vaccination, with a total of 112 doses. Most patients (22/30 [73%]) received MMR vaccination followed by VAR (19/30 [63%]), BCG (18/30 [60%]), rotavirus (11/30 [37%]), and LJEV (9/30 [30%]) vaccinations. In group C, the MMR vaccination coverage rate was the highest (70% [39/56]), followed by the coverage rates of VAR (41% [23/56]), BCG (41% [23/56]), rotavirus (20% [11/56]), and LJEV (11% [6/56]) vaccinations.

### 3.3. AEFLI

**Figure 2:**
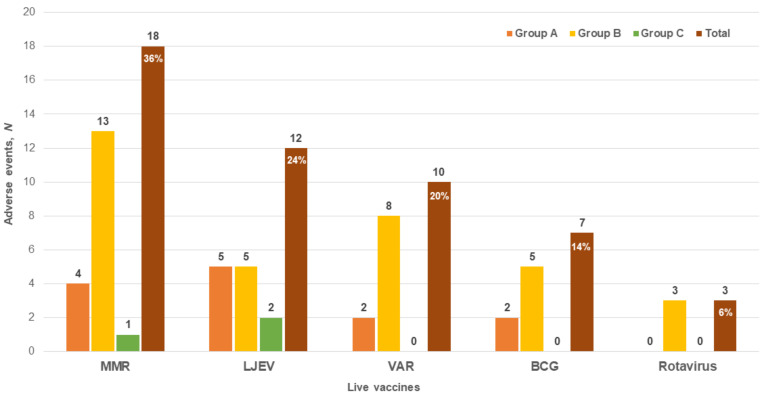
Adverse events following live immunization (n = 50). Abbreviations: BCG, Bacillus Calmette–Guérin; MMR, measles-mumps-rubella; VAR, live-attenuated varicella; LJEV, live-attenuated Japanese encephalitis virus.

Among 73 recipients of live vaccines, 17 showed a total of 50 AEFLI. The MMR, LJEV, VAR, BCG, and rotavirus vaccines were associated with 36%, 24%, 20%, 14%, and 6% AEFLI, respectively (Figure 2). AEs included fever (*n* = 29), upper respiratory infection (*n* = 9), diarrhea (*n* = 4), rash (*n* = 3), thrombocytopenia (*n* = 3), injection site pus (*n* = 1), and febrile convulsion (*n* = 1).

In group A, 13 AEFLI (LJEV, 5; MMR, 4; VAR, 2; BCG, 2) occurred after 29 live vaccine doses and in group B, 34 AEFLI (MMR, 13; VAR, 8; BCG, 5; LJEV, 5; rotavirus, 3) occurred after 112 live vaccine doses. No significant difference was observed in the incidence of AEs between groups A and B (45% vs. 30%, *p* = 0.14) (Table 3).

Applying the WHO 2019 causality assessment algorithm [24] to the 50 events, 12%, 58%, and 30% were categorized as consistent with live vaccination, indeterminate, and inconsistent, respectively. Moreover, serious AEs, such as intensive care unit hospitalization, death, and emergence of diseases caused by vaccine strains, were not observed.

## 4. Discussion

To the best of our knowledge, this is the first study to investigate AEFLI in patients with DGS in Korea. In addition to MMR and VAR vaccines, AEs after BCG, rotavirus, and LJEV vaccinations, which were rarely studied in the past, were also evaluated. Approximately 60% of patients with DGS received live vaccines without T cell function evaluation. Nevertheless, live vaccines were found to be generally well tolerated and safe in the present study, even in patients with moderate T cell deficiency.

Since the 2010s, the IDSA, AAP, and Korean Pediatric Society have recommended MMR and VAR vaccines for consideration in patients with pDGS with adequate T cell counts (≥500 CD3 T cells/mm^3^ and ≥200 CD8 T cells/mm^3^) and normal mitogen response [17,18,21]. Previous cohort studies have shown that unvaccinated individuals with pDGS experienced mild-to-moderate live vaccine-preventable illnesses [16,25]. Further, several studies have reported high rates of VAR and measles infections among unvaccinated patients, reflecting increased disease exposure and decreased herd immunity [14,26]. Therefore, live vaccines are beneficial in patients with pDGS with reasonable T cell function. However, depending on the degree of T cell deficiency, live vaccines can induce disease via the viral strains; hence, it is essential to evaluate T cell immunity before live vaccine inoculation. In the present study, T cell immunity status was evaluated in only approximately 40% of patients with DGS. Among patients who underwent the T cell test, 21% showed moderate deficiency (group A), which is currently contraindicated for live vaccines; however, no serious AEs, including infection by vaccine strains, were observed. This could be due to normal T cell function despite moderate deficiency. Although we did not test mitogen response (a test to evaluate T cell function), previous studies have reported normal mitogen response in patients with pDGS [15,25]. Nevertheless, further studies are needed to ascertain live vaccine safety in patients with DGS after thorough evaluation of mitogen response and CD3 and CD8 T cell immunity. Our study emphasized the need for immune screening before live vaccination in children diagnosed with DGS.

Most studies evaluating AEFLI in patients with pDGS have only been conducted for MMR and VAR vaccinations and demonstrate coverage rates of 47–88% and 25–75% [14,16,25], respectively. Further, AEFLI rates associated with MMR and VAR vaccinations are 7–22% and 20%, respectively, with no reported serious AEs [14,15,16]. In Korea, two MMR vaccine doses are administered at 1 year and 4–6 years of age, and VAR is inoculated at 1 year of age. In the present study, we observed that MMR vaccination coverage was the highest (70%), and the AE rates after MMR and VAR vaccinations were 18% (18/101) and 19% (10/53), respectively, with no serious AEs; these findings were consistent with those of previous studies.

Studies reporting AEFLI for BCG, rotavirus, and LJEV vaccines are limited. Although previous studies aimed to analyze AEs by including patients who received BCG and rotavirus vaccines, the sample sizes were small, and analyses of AEFLI were not sufficient [16,27]. In the Korean national mandatory vaccination schedule, BCG and Japanese encephalitis vaccines are administered within 4 weeks of age and after 1 year of age, respectively. For Japanese encephalitis, both live and inactivated vaccines are available. In addition, the rotavirus vaccine is selectively provided at 2, 4, and 6 months (for RotaTeq^®^) or 2 and 4 months (for Rotarix^®^) of age, and according to Korean national data in 2017, the rotavirus vaccine coverage rate was >85% [20,21]. The Korean vaccination schedule provided us with a unique opportunity to evaluate the AEs of live vaccines, such as BCG, rotavirus, and LJEV vaccines, which are rarely evaluated in patients with DGS. In the present study, although the live vaccine coverage rates were lower than the rates in the general Korean population [19], the number of participants was larger than that in previous studies. The rates of AEs following BCG, rotavirus, and LJEV vaccines were 16% (7/45), 6% (3/53), and 35% (12/34), respectively. Most symptoms were mild, and no serious AEs were identified. In group A, seven mild AEs were observed among the recipients of LJEV (5) and BCG (2) vaccinations, with fever being the most common (4/7). According to the 2019 WHO causality assessment algorithm, only one event—fever—was consistent with live vaccination in group A. Surveillance data for AEFI from 2011 to 2016 in South Korea showed that the average AEs reporting rate of BCG, MMR, VAR, and LJEV were 19, 1.7, 1.7, and 0.4 cases/100,000 doses, respectively [28]. Since it was a passively collected reporting system, there would have been many cases of missed reporting, and the AEFI reporting rates were lower than our results. However, while serious AEs including vaccine strain infection (BCG osteomyelitis, 4; disseminated BCG infection, 1; soft tissue infection, 1; infectious arthritis, 1; anaphylactic reaction, 1) have been reported in the surveillance system [28], there were no serious AEs in our study. Our results suggest that the current guidelines for MMR and VAR vaccines in patients with pDGS can also be applied to BCG, rotavirus, and LJEV vaccines.

This study has some limitations. First, it was a retrospective, single-center study; hence, it is inherently limited due to the non-random distribution of the study participants. Further, the sample size was relatively small. Second, immune screening data were lacking, which may have resulted in overestimation of immune status. Finally, we estimated AEFLI from medical visit records; this may have resulted in fewer AEFLI being identified and limited data for causality assessment. Nevertheless, our study analyzed the AEFLI of a wide range of live vaccines (BCG, rotavirus, LJEV, MMR, and VAR) simultaneously, in a population of patients with DGS.

## 5. Conclusions

In conclusion, our data indicated that live immunizations are well-tolerated by patients with pDGS and emphasized that immune screening tests should be encouraged before all live vaccinations in patients with DGS. Furthermore, this study of the safety of live vaccine immunization may contribute to alleviating vaccine hesitancy and facilitating greater coverage among patients with immunodeficiency. In this study, there was a lack of immune status evaluation data at the time of vaccination in patients with DGS. The evaluation of side effects, combined with T cell function evaluation in patients with DGS, would help us to obtain a better profile of the safety of live vaccination in that population. Additional multicenter, prospective, large-scale studies are required to validate our findings and provide further guidance for live immunizations in patients with DGS.

## Figures and Tables

**Table 1:** Characteristics of the study population.

Characteristics	*N* (%) or Median (IQR)
**Sex (Male), *n* (%)**	51 (54.3)
**Age of disease confirmation, median (IQR), month**	7.9 (0.7–104.3)
**Chromosome 22q11.2 deletion, *n* (%)**	94 (100)
FISH	54 (57.4)
MLPA	26 (27.7)
Others	14 (14.9)
**Cardiac disease, *n* (%)**	69 (73.4)
Ventricular septal defect	25 (36.2)
Tetralogy of Fallot	22 (31.9)
Interrupted aortic arch	9 (13.0)
Others	13 (18.8)
S/p operation	61 (88.4)
Hypocalcemia, *n* (%)	30 (31.9)
Facial dysmorphism, *n* (%)	50 (53.2)
Developmental delay, *n* (%)	38 (40.4)
Epilepsy, *n* (%)	6 (6.4)
Schizophrenia, *n* (%)	2 (2.1)
Death, *n* (%)	5 (5.3)
**No. of lymphocyte subsets test, *n* (%)**	38 (40.4)
Age at test, median (IQR), month	2.8 (1.0–75.5)
**CD3 ≥ 500 and CD8 ≥ 200 (cells/mm^3^), *n* (%)**	30 (79)
CD3 (cells/mm^3^), median (range)	1425 (536–4234)
CD8 (cells/mm^3^), median (range)	421 (242–2001)
CD4 (cells/mm^3^), median (range)	938 (231–3001)
**CD3 < 500 or CD8 < 200 (cells/mm^3^), *n* (%)**	8 (21)
CD3 (cells/mm^3^), median (range)	378 (205–497)
CD8 (cells/mm^3^), median (range)	179 (35–198)
CD4 (cells/mm^3^), median (range)	245 (139–345)
**<50 CD3 (cells/mm^3^), *n* (%)**	0 (0)
**Mitogen response, *n* (%)**	2 (2.1)
>10	1 (50)
≤10	1 (50)
**Thymus screening, *n* (%)**	28 (29.7)
Hypoplasia or absent	11 (39.3)
Normal	17 (60.7)

Abbreviations: FISH, fluorescence in situ hybridization; MLPA, multiple ligation probe amplification; IQR, interquartile range.

**Table 2:** Live immunization coverage in the study population.

	Group A (*n* = 8) ^a^		Group B (*n* = 30) ^b^		Group C (*n* = 56) ^c^		Total (*n* = 94)	
	Immunized Patients, *N* (%)	Vaccine Doses (*N*)	Immunized Patients, *N* (%)	Vaccine Doses (*N*)	Immunized Patients, *N* (%)	Vaccine Doses (*N*)	Immunized Patients, *N* (%)	Vaccine Doses (*N*)
BCG	4 (50)	4	18 (60)	18	23 (41)	23	45 (48)	45
Rotavirus	1 (13)	2	11 (37)	25	11 (20)	26	23 (24)	53
MMR	5 (63)	10	22 (73)	32	39 (70)	59	66 (70)	101
VAR	5 (63)	7	19 (63)	20	23 (41)	26	47 (50)	53
LJEV	3 (38)	6	9 (30)	17	6 (11)	11	18 (19)	34
Total ^d^	6 (75)	29	24 (80)	112	43 (77)	145	73 (78)	286

Abbreviations: BCG, Bacillus Calmette–Guérin; MMR, measles–mumps–rubella; VAR, live-attenuated varicella; LJEV, live-attenuated Japanese encephalitis virus. ^a^ Patients with moderate T cell deficiency, CD3 < 500 or CD8 < 200 cells/mm^3^. ^b^ Patients with mild-to-normal T cell deficiency, CD3 ≥ 500 and CD8 ≥ 200 cells/mm^3^. ^c^ Patients without lymphocyte subset results. ^d^ The number of patients who received at least one live immunization in each group.

**Table 3:** Adverse events following live immunization and causality assessments.

Group ^a^	Sex	Age (Months)	Vaccination Type	Adverse Events	Interval Day(s) ^b^	Algorithm ^c^	Causality 2019 ^c^	Causality 1999 ^d^
A	F	96	LJEV	fever	1	consistent	A1	very likely
A	M	13	LJEV	fever (UTI)	35	inconsistent	C	unlikely
A	M	13	LJEV	cough	35	indeterminate	B2	unlikely
A	F	12	MMR	fever	8	indeterminate	B1	unlikely
A	F	12	MMR	diarrhea	8	indeterminate	B2	possible
A	F	12	VAR	diarrhea	8	indeterminate	B2	unlikely
A	F	12	VAR	fever	8	indeterminate	B1	possible
A	F	12	LJEV	fever	8	indeterminate	B1	possible
A	F	12	LJEV	diarrhea	8	indeterminate	B2	unlikely
A	F	3	BCG	fever (rhinovirus)	15	inconsistent	C	unrelated
A	F	3	BCG	cough (rhinovirus)	15	inconsistent	C	unrelated
A	M	12	MMR	fever	19	indeterminate	B1	unlikely
A	M	12	MMR	cough	19	indeterminate	B2	unlikely
B	M	3	BCG	injection site pus ^e^	47	consistent	A1	very likely
B	F	1	BCG	rash	94	indeterminate	B2	possible
B	F	12	MMR	fever	1	consistent	A1	very likely
B	F	13	MMR	fever	8	indeterminate	B1	possible
B	M	59	MMR	fever	19	indeterminate	B2	unlikely
B	F	16	MMR	cough	28	indeterminate	B2	unlikely
B	F	16	MMR	fever	28	indeterminate	B2	unlikely
B	F	12	VAR	fever	0	consistent	A1	very likely
B	F	13	VAR	fever	8	indeterminate	B1	possible
B	M	12	VAR	fever	13	indeterminate	B2	unlikely
B	M	28	LJEV	fever	2	consistent	A1	very likely
B	M	16	LJEV	febrile convulsion ^f^	51	indeterminate	B2	unlikely
B	M	0	BCG	fever (UTI)	52	inconsistent	C	unrelated
B	F	6	BCG	fever (RSV)	119	inconsistent	C	unrelated
B	F	6	BCG	cough (RSV)	119	inconsistent	C	unrelated
B	M	7	Rota	fever (UTI)	37	inconsistent	C	unrelated
B	F	7	Rota	cough	49	indeterminate	B2	unlikely
B	F	7	Rota	fever	49	indeterminate	B2	unlikely
B	M	12	MMR	fever	13	indeterminate	B2	unlikely
B	M	12	MMR	cough (RSV)	25	inconsistent	C	unrelated
B	M	12	MMR	fever (RSV)	25	inconsistent	C	unrelated
B	M	13	MMR	thrombocytopenia ^g^ (post cardiac operation condition)	30	inconsistent	C	unrelated
B	F	12	MMR	rash	33	indeterminate	B2	probable
B	F	12	MMR	thrombocytopenia	33	indeterminate	B2	probable
B	F	12	MMR	fever	33	indeterminate	B2	unlikely
B	M	56	MMR	fever (rhinovirus)	48	inconsistent	C	unlikely
B	M	12	VAR	cough (RSV)	25	inconsistent	C	unrelated
B	M	12	VAR	fever (RSV)	25	inconsistent	C	unrelated
B	F	12	VAR	rash	33	indeterminate	B1	probable
B	F	12	VAR	thrombocytopenia	33	indeterminate	B2	unlikely
B	F	12	VAR	fever	33	indeterminate	B2	unlikely
B	M	25	LJEV	fever	21	inconsistent	C	unrelated
B	F	28	LJEV	cough	21	indeterminate	B2	unlikely
B	M	16	LJEV	fever	51	inconsistent	C	unrelated
C	M	13	MMR	fever	8	indeterminate	B1	unlikely
C	M	27	LJEV	fever	1	consistent	A1	very likely
C	M	27	LJEV	diarrhea	1	indeterminate	B1	probable

Abbreviations: BCG, Bacillus Calmette–Guérin; MMR, measles-mumps-rubella; VAR, live-attenuated varicella; LJEV, live-attenuated Japanese encephalitis virus; M, male; F, female; UTI, urinary tract infection; RSV, respiratory syncytial virus. ^a^ Categorized by results of lymphocyte subsets; group A [CD3 ≥ 500 and CD8 ≥ 200 (cells/mm^3^)], group B [CD3 < 500 or CD8 < 200 (cells/mm^3^)], and group C without lymphocyte subset results. ^b^ The number of days between the date of live immunization and the occurrence of adverse events. ^c^ WHO causality assessment, 2019; A1, vaccine product-related reaction; B1, temporal relationship is consistent but there is insufficient definitive evidence for vaccine causing event; B2, qualifying factors result in conflicting trends of consistency and inconsistency with causal association to immunization; C, coincidental underlying or emerging condition(s), or condition(s) caused by exposure to something other than vaccine. ^d^ WHO Immunization safety surveillance, 1999.^.e^ No systemic symptoms and no requirement of systemic antibiotics. ^f^ Simple febrile convulsion; no need for anti-epileptic drugs or hospitalization. ^g^ Platelet counts were <150,000/μL and >50,000/μL in all cases.

## Data Availability

Not applicable.
